# The synthesis of *N*,1,4-tri(alkoxy-hydroxybenzyl)-1,4-diazepane-amines: investigations on reaction characteristics and mechanism

**DOI:** 10.1098/rsos.240293

**Published:** 2024-07-03

**Authors:** Anne Zenner, Johannes Steinmetzer, Nico Ueberschaar, Martin Freesmeyer, Wolfgang Weigand, Julia Greiser

**Affiliations:** ^1^ Working Group for Translational Nuclear Medicine and Radiopharmacy, Clinic of Nuclear Medicine, Jena University Hospital, Jena, Germany; ^2^ Institute of Physical Chemistry and Abbe Center of Photonics, Friedrich Schiller University Jena, Jena, Germany; ^3^ Mass Spectrometry Platform, Friedrich Schiller University Jena, Jena, Germany; ^4^ Institute for Inorganic and Analytical Chemistry, Friedrich Schiller University Jena, Jena, Germany

**Keywords:** 1,4-diazepane-6-amine, reductive amination, synthesis investigation

## Abstract

1,4-Diazepane-6-amine (DAZA) can be alkylated with three 2-hydroxybenzyl pendant arms, resulting in hexadentate chelators suitable for coordination of radiometals like ^68^Ga. These chelators, *N*,1,4-tri(alkoxy-2-hydroxybenzyl)-DAZA, can be produced via a one-pot synthesis, with the first step being a carbonyl amine condensation of DAZA with two respective 4-alkoxy-2-hydroxybenzaldehydes, followed by reductive amination with sodium borohydride. While the first step of this reaction is predictable, the subsequent reductive amination can result in either mono-, di- or tri(alkoxy-hydroxybenzyl)-DAZA compounds. Seeking to identify dependencies that might allow a specific reaction control towards the formation of either of the three possible products, and particularly towards the favoured trialkylated DAZA compounds, a variety of synthesis trials were performed. Additionally, computational methods were employed to evaluate the underlying reaction mechanism. Synthesis trials verified that the trialkylated DAZA compounds are formed via direct reductive amination of the dialkylated DAZA compounds. Subsequently, a synthetic method was established, leading to an increase in the percentage of the trialkylated DAZA compounds, which allowed the successful isolation of those hexadentate chelators. Additionally, an alternative pathway proceeding via aminal C–N bond insertion of an attacking third carbonyl moiety was evaluated by means of quantum chemical calculations but so far remains entirely hypothetical.

## 1. Introduction

*N*,1,4-Tri(4-alkoxy-2-hydroxybenzyl)-1,4-diazepan-6-amines (TAoS-DAZA) are a novel group of heterocyclic chelators [1]. These ligands combine three nitrogen atoms on the flexible azacycle 1,4-diazepan-6-amine (DAZA) [2] with three phenolate pendant arms serving as oxygen donors, thus to a certain degree being comparable to salen chelators or previously described hydroxybenzyl triazacyclononanes [3–5]. As hexadentate ligands, the TAoS-DAZA chelators exhibit great potential to bind a variety of metal ions, among which the trivalent Ga(III) ion is of particular interest. Its radioactive isotope ^68^Ga serves as the basis for a growing variety of radiopharmaceuticals, which are used as imaging agents in positron emission tomography (PET) [6]. Over the course of the last two decades, PET has emerged as a powerful, non-invasive tool in the field of nuclear medicine for the diagnosis of cancerous and inflammatory diseases as well as a provider of functional or metabolic information [7,8]. The last couple of years have seen a major surge in new radiopharmaceuticals based on ^68^Ga because of its steady supply from portable, long-lived ^68^Ge/^68^Ga generators, which allow on-site radiopharmaceutical production without requiring the immediate proximity to a cyclotron [6]. The half-life of ^68^Ga (68 min) is sufficiently long to allow radiopharmacists to perform the essential steps of clinical routine production, which usually includes the chelation of the radiometal with a suitable ligand and subsequent purification [7,9]. At the same time, the half-life of ^68^Ga is adequately short to prevent long-term radiation exposure of patients and personnel [6,10]. By now a wide range of specifically designed bifunctional ligands are available, making an equally broad spectrum of ^68^Ga radiopharmaceuticals with different fields of application accessible [10–12]. Our group has designed and established the ^68^Ga chelator group TAoS-DAZA as a potential hepatobiliary PET imaging agent [1,13]. Recently, we could show that the [^68^Ga]Ga-TAoS-DAZA complexes are suitable for liver imaging with PET, allowing for biliary stent integrity diagnosis, potentially also for liver function quantification and differential diagnosis of liver nodules [14,15].

So far, two derivatives of TAoS-DAZA have been synthesized, characterized and studied *in vivo*, namely *N*,1,4-tri(4-methoxy-2-hydroxybenzyl)-DAZA (TMoS-DAZA, also formerly known as TMeOHB-DAZA) and *N*,1,4-tri(4-ethoxy-2-hydroxybenzyl)-DAZA (TEoS-DAZA, also formerly known as TEtOHB-DAZA). Both ligands can be produced via a one-pot synthesis, with the first step being a carbonyl amine condensation of DAZA with the respective 4-alkoxy-2-hydroxybenzaldehyde, followed by reductive amination with sodium borohydride as the second step [1]. The mechanism of the first reaction step is easily understood and features the introduction of one carbonyl moiety through Schiff base formation at the primary amino group of DAZA, and of a second carbonyl moiety by forming a bisaminal bridge between the two secondary nitrogen atoms of the DAZA azacycle [1,16–21]. The subsequent reaction step, however, seemingly involves an additional nitrogen atom alkylation, thus resulting in trialkylated chelators (TAoS-DAZA), although no obvious additional alkylating agent but only sodium borohydride is added to the reaction [1]. In addition, this initially counterintuitive reaction, which turns a compound carrying two hydroxybenzyl moieties into a compound carrying three hydroxybenzyl moieties, even delivers those trialkylated DAZA compounds in appreciable yields [1]. Therefore, the underlying mechanism of this reductive amination reaction merits investigation.

Aiming to synthesize more novel derivatives of the TAoS-DAZA family, we initially encountered problems during the reductive amination regarding the isolation of the desired trialkylated DAZA ligand. Instead of the expected formation and precipitation of the desired trialkylated DAZA compound, we repeatedly observed that a byproduct was formed in substantial percentages, which we ultimately identified to be a dialkylated DAZA substrate.

We sought to investigate the cause for these differences in reaction specificity regarding the formation of trialkylated versus dialkylated DAZA, ultimately aiming to understand the factors promoting the additional alkylation step. For this purpose, synthesis trials and additional quantum chemical methods were used to identify a probable reaction mechanism. Ultimately, the findings from these studies will allow a more specific reaction control towards the isolation of novel trialkylated DAZA ligands. Once successful, thereby it would be possible to create a new generation of ligands based on the DAZA heterocycle.

## 2. Material and methods

**Figure 1.**
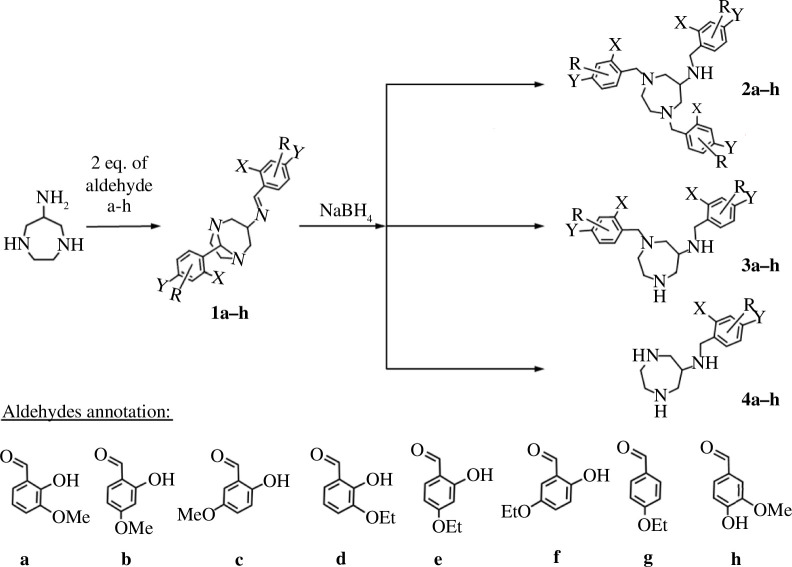
The possible reaction pathways of the reductive amination of **1**. Depending on the reaction parameters and the substitution pattern (R*, X and Y*), tri- (**2**), di- (**3**) and monoalkylated (**4**) DAZA compounds are formed. Substitution patterns are labelled as follows: **a**: X = OH, R = 3 OMe; **b**: X = OH, R = 4 OMe; **c**: X = OH, R = 5 OMe; **d**: X = OH, R = 3 OEt; **e**: X = OH, R = 4 OEt; **f**: X = OH, R = 5 OEt; **g**: X = H, R = 4 OEt; **h**: X = H, R = 3 OMe, Y = OH.

The aromatic substitution patterns **a**–**h** of the compounds investigated herein correspond to those of the respective benzaldehyde that was used for the reductive amination of the DAZA azacycle, which are attributed as follows (figure 1): **a**: X = OH, R = 3 OMe; **b**: X = OH, R = 4 OMe; **c**: X = OH, R = 5 OMe; **d**: X = OH, R = 3 OEt; **e**: X = OH, R = 4 OEt; **f**: X = OH, R = 5 OEt; **g**: X = H, R = 4 OEt; **h**: X = H, R = 3 OMe, Y = OH.

All chemicals were purchased from commercial suppliers and used without any further purification. High-resolution electro-spray mass spectra (ESI-MS) were obtained using a Bruker maXis mass spectrometer using direct infusion. HPLC-MS was measured with an Agilent 1100 HPLC, column: Gemini^®^ NX-C18, and a Thermo LTQ mass spectrometer. Solvents were A: 2% acetonitrile (ACN), 0.1% formic acid in water and B: 100% ACN, flow rate: 0.5 ml min^−1^, gradient: 5–95% B over the course of 10 min; ultraviolet absorption channel A: 220 nm, channel B: 254 nm, channel C: 280 nm. Mass spectra were measured in positive and negative modes between 150 and 1500 *m*/*z*. In addition to LC-MS, analysis of reaction mixtures was performed with HPLC on an HP 1100 instrument (Agilent) using a Eurosphere 125 × 4 mm column (100-5 C18, Knauer) and the following gradient: 0.0–2.5 min 97.0% A, 2.5–10.0 min 97.0% A → 0.0% A, 10.0–13.0 min 0.0% A, 13.0–13.05 min 0.0% A → 97.0% A, 13.05–16.0 min 97.0% A, A being water/trifluoroacetic acid (99.9/0.1 v/v) and being acetonitrile/trifluoroacetic acid (99.9/0.1 v/v). NMR spectra were recorded at 400 MHz using a Bruker Avance III.

### 2.1. Synthesis of bicyclic bisaminal compounds 1a–h

To a methanolic solution containing 1 equivalent (eq.) of DAZA, 2 eq. of the respective benzaldehyde **a**–**h** were added. The mixture was stirred for 1 h at room temperature (RT) and the yellow residue was removed by filtration, washed with methanol and dried *in vacuo*. In the case of **1g** the product formed colourless crystals upon partial solvent evaporation, and the crystals were isolated via filtration and washed with cold diethyl ether.

**Yields: 1b**: 94% [1], **1c**: 95%, **1e**: 94% [1], **1f**: 77%, **1g**: 85%.

A yield for **1a**, **1d** and **1h** could not be determined since there was no precipitation. Therefore, the raw reaction mixture was used for the subsequent reductive amination. Synthesis protocol, analysis and yields for **1b** and **1e** were already reported in a previous publication [1]. Detailed synthesis protocols are given in the electronic supplementary material.

### 2.2. General procedure for reductive amination (‘single addition’)

One eq. of **1a**–**1h** was suspended in a mixture of methanol and chloroform (1/1, v/v; **1a**–**1c**, **1h**) or in methanol (**1d**–**1f**, **1g**), to which 2 eq. of NaBH_4_ were added. The mixture was stirred overnight at RT, after which solvents were removed under reduced pressure. The residue was dissolved again in methanol to stimulate precipitation. However, precipitation only occurred during the reduction of **1e**, yielding **2e** as a pure precipitate. In all other cases, the raw reaction mixture was analysed by LC-MS.

### 2.3. Separation and identification of reaction mixture 2b/3b

Column chromatography was performed for the raw mixture of **2b** and **3b** according to the gradient listed in table 1, using silica gel. Each fraction contained a volume of 8 ml (except fraction 1). Compound **3b** could be found in fractions 13–18 and was characterized via NMR.

**Table 1.** Solvents and fractions used for preparative HPLC of reaction mixture **2b** and **3b**.

used solvents	volume (ml)	number of fractions
chloroform/hexane 1:1 + 1% triethylamine	75	1 (50 ml)–5
chloroform/hexane 2:1 + 1% triethylamine	50	5–11
chloroform/triethylamine 100:1	100	12–23
chloroform/triethylamine/methanol 100:1:1.5	75	24–33
chloroform/triethylamine/methanol 100:1:3	50	34–39
chloroform/triethylamine/methanol 100:2:10	100	40–51
methanol/triethylamine 100:1	80	52–60

### 2.4. Procedure for reductive amination leading to isolation of the trialkylated compounds 2a–2h (‘multi-addition’)

The reaction mixture gained from the first reductive amination step (single addition) was resuspended in a mixture of methanol and chloroform (1/1, v/v; **a**–**c**, **h**) or in methanol (**d**–**f**) and 1 eq. of the respective aldehyde **a**–**h** was added. After allowing to stir for 5–10 min to realize complete aldehyde dissolution and homogenization, 1 eq. of NaBH_4_ was added and the mixture was stirred at RT overnight. Subsequently, the procedure was repeated up to two more times by again adding 1 eq. of aldehyde and NaBH_4_ subsequently and stirring overnight. The resulting precipitates **2a**–**2f** and **2h** were removed byfiltration and washed with methanol and diethyl ether. Detailed synthesis protocols for each compound are given in the electronic supplementary material. NMR and MS results of the products are reported in the electronic supplementary material with the exception of data for **2b** and **2e**, since the characterization for those compounds has already been published [1]. Compound **2g** could not be isolated in sufficient purity, thus not allowing for yield determination and NMR analysis of the compound. Nevertheless, the formation of **2g** was suggested in the LC-MS (electronic supplementary material).

**Yields: 2a**: 94%, **2b**: 85%, **2c**: 66%, **2d**: 73%, **2e**: 88%, **2f**: 79%, **2h**: 43%.

### 2.5. Synthesis of 4g

To a methanolic solution (50 ml) containing 1 eq. (268 mg, 2.33 mmol) of DAZA, 2 eq. (700 mg, 4.66 mmol) of 4-ethoxybenzaldehyde (**g**) were added. The mixture was stirred for 24 h at RT after which the volume of the solvent was reduced from 50 to 5 ml under reduced pressure and the colourless residue was removed by filtration to afford **1g** (750 mg, 1.98 mmol, 85%).

Compound **1g** (980 mg, 2.6 mmol) was dissolved in methanol (30 ml) and NaBH_4_ (220 mg, 5.8 mmol) was added. The mixture was stirred at RT for 2 h. Subsequently, aqueous hydrochloric acid (4.0 M, 1 ml) was added and the solvent was removed under reduced pressure. The residue was suspended in ethanol, filtered, washed two times with ethanol and two times with chloroform and was identified as the hydrochloride of **4g**. The colourless precipitate **4g** was dissolved in water (10 ml) and sodium hydroxide (1.0 M) was added dropwise until the pH value reached 9. The aqueous phase was extracted four times using chloroform, the organic phases were united, dried over Na_2_SO_4_ and the solvent was removed under reduced pressure to yield **4g** as a colourless oil (460 mg, 1.85 mmol, 71%).

### 2.6. Quantum chemical calculations

Quantum chemical calculations (QCC) were carried out using density functional theory (DFT). All energies and their derivatives were calculated using ORCA 5.0.4 [22], which was interfaced with the external optimizer pysisyphus [23]. The viability of the different reaction paths was compared by calculating the differences in Gibbs free energies (barrier heights) between educts and the associated transition states. Two different routes towards the formation of **2e** were investigated: (a) direct reductive amination of dialkylated **3e** and (b) insertion of a carbonyl into the aminal bridge of **1e**. The electron flow during the carbonyl insertion into **1e** was interpreted in terms of intrinsic bond orbitals (IBOs) [24,25]. A detailed computational protocol is given in the electronic supplementary material [26–30].

## 3. Results

### 3.1. Competing products are formed during the reductive amination when employing the single addition procedure

In contrast to the previously reported isolation of **2b** (TMoS-DAZA) and **2e** (TeoS-DAZA), which featured precipitation of the desired trialkylated products [1], no precipitation of the related 2-hydroxy-bearing compounds **2a**–**2d** and **2f** was observed using the described ‘single addition’ procedure. Consequently, the trialkylated chelators **2a**–**2d** and **2f** could not be isolated by simple filtration, the only exception being the 4-ethoxy-bearing derivative (**2e**), which could be produced according to our previously published protocol [1].

**Figure 2.**
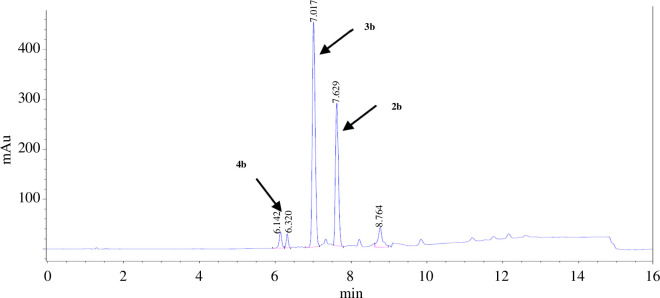
Representative HPLC chromatogram of a mixture of **2b** (7.6 min), **3b** (7.0 min) and **4b** (6.1 and 6.3 min). Monoalkylated **4b** gives two peaks, indicating isomers bearing the phenolate pendant arm at the primary or a secondary nitrogen atom of the DAZA azacycle.

**Figure 3.**
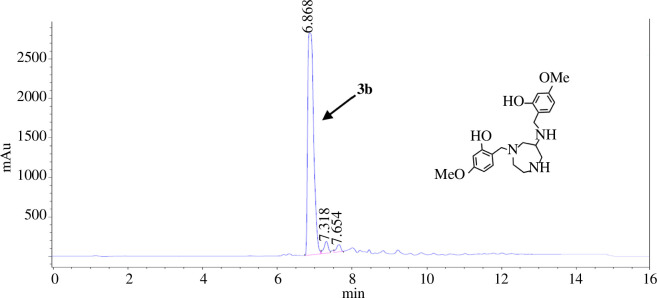
HPLC chromatogram of the isolated byproduct **3b** (6.9 min).

HPLC analysis of the raw reaction mixtures after the single addition procedure consistently revealed the presence of a respective byproduct **3a**–**3f** in addition to the desired trialkylated products **2a**–**2f** (figure 2). We suspected **3a**–**3f** to be dialkylated substrates, resulting from reductive cleavage of the aminal bridge in **1a**–**1f**, thereby forming one secondary and one tertiary nitrogen atom within the azacycle, as well as reduction of the imine moiety at the exocyclic nitrogen atom (figure 1). This reaction and similar dialkylated products have previously been described by Neis *et al*. [19]. To verify our assumption, we aimed to isolate the substrates **3**. Since separating and isolating **2a**–**2f** and **3a**–**3f** by means of recrystallization proved unsuccessful in all cases, we performed preparative HPLC on the reaction mixture of **2b** and **3b** as representative substrates (figure 2). Indeed, NMR spectroscopic investigation and MS analysis verified the structure of **3b** (figure 3). In addition to the trialkylated and the dialkylated products **2a**–**2f** and **3a**–**3f**, HPLC analysis also revealed the presence of the monoalkylated substrates **4a**–**4f**, albeit in low percentages of ≤6%.

Results similar to those for **a–f** were observed when using aldehydes **g** and **h**. Likewise, LC-MS analysis revealed the presence of dialkylated and trialkylated compounds **2g**–**2h** and **3g**–**3h**. Notably, from a large-scale reductive amination of **1g** the monoalkylated product **4g** was isolated as the major product. Compound **4g** serves as a representative compound for the monoalkylated byproducts, which in cases of **4a**–**4f** and **4h** were present in percentages too low to merit respective product isolation.

**Figure 4.**
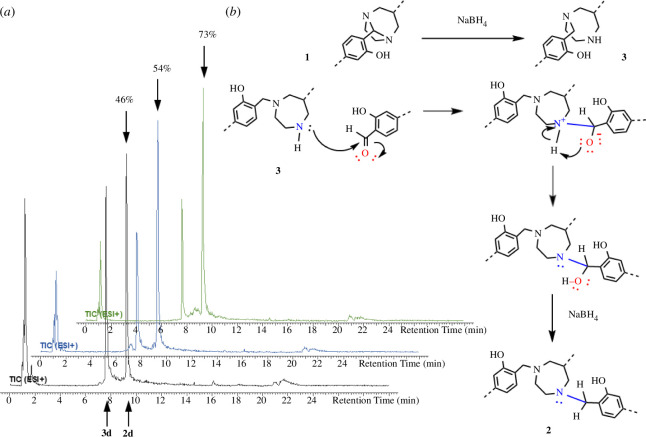
(*a*) LC chromatograms from LC-MS analysis, showing the increase of trialkylated product **2d** (9.3 min) from 46% (black) to 54% (blue) to 73% (green) after each renewed addition of aldehyde and NaBH_4_ (multi-addition). The percentages do not indicate true percentages in the reaction solution, as ultraviolet–visible absortion characteristics are not taken into account, but are solely meant to visualize the increasing amount of **2d** in the samples. Injection peaks have not been included in the calculations. (*b*) Reaction scheme depicting the formation of trialkylated **2** via direct reductive amination of **3**. At first, **3** is formed as a result of aminal bond reduction of **1**. Then, an attacking carbonyl reacts with **3** in a direct reductive amination. The barrier height for the entire reaction (calculated for **3e** → **2e**) was 138 kJ mol^−1^. The reaction occurs via an initial nucleophilic attack of the amine-nitrogen on the carbonyl-carbon, during which the CN distance is substantially decreased. Then, the H-transfer from the amine onto the carbonyl-oxygen occurs, forming a hemiaminal prone to reduction with NaBH_4_, resulting in **2**.

While the formation of the dialkylated substrates **3** requires only a reductive cleavage of the aminal bridge (figure 4), the formation of the trialkylated DAZA compounds **2** requires an additional reductive *N*-alkylation at the DAZA heterocycle, likely by a free carbonyl component (**a**–**h**) resulting from *in situ* hydrolysis of compounds **1**. This carbonyl moiety is then attached to the secondary nitrogen atom, likely via an iminium species, and subsequently reduced, thus turning dialkylated **3** into trialkylated **2** (figure 4). Consequently, in a reaction mixture with a limited amount of free carbonyl, the formation of **2** can only occur at the price of partial hydrolysis of **1**, releasing a carbonyl moiety which then attacks another molecule **3**. However, if substantial amounts of **1** are instead straightforwardly reduced to **3**, the lack of free carbonyl moieties logically should reduce the percentage of **2**. That means, ultimately **2** and **3** can be considered competing products of this reductive amination reaction.

It became apparent that the reductive amination of **1a**–**1f** in all cases resulted in a mixture of predominantly **2** and **3** in varying percentages. Exact quantification from the LC-MS and HPLC chromatograms was prohibited since the ultraviolet–visible absorption characteristics of substrates **2a**–**2f** and **3a**–**3f** are not yet known; however, the reaction mixtures from the first reductive amination trial often exhibited higher signal intensity for the dialkylated compound **3**, rather than **2** (figure 2).

### 3.2. Reductive amination of 3 leads to trialkylated compound 2 when applying the multi-addition procedure

We discovered that repeatedly adding fresh NaBH_4_ and aldehyde to the product mixture gained after the single addition procedure led to an increase in the percentage of the trialkylated products **2a**–**2h**, especially after repeating the process of aldehyde/NaBH4 addition up to three times (‘multi-addition’). The progress of increasing formation of **2** could be monitored via LC-MS after each repeated reduction step, as shown representatively for **2d** (figure 4). This procedure allowed successful isolation of the trialkylated compounds **2a**–**2f** and **2h** in yields of up to 94%, because the increase of the percentage of **2a**–**2f** and **2h** led to product precipitation suitable for filtration and subsequent characterization of **2a**–**2f** and **2h** (see electronic supplementary material).

The findings from this multi-addition procedure verify that dialkylated DAZA compound **3** can be turned into **2** via direct reductive amination at the secondary endocyclic nitrogen atom, as long as there is sufficient free carbonyl component for the reaction to occur (figure 4).

### 3.3. Some observations regarding the influence of the aromatic substitution pattern on reaction specificity

We observed an influence of the reaction solvent on the performance of the reductive amination. Namely, the reductive amination of methoxy-bearing substrates **1a**–**1c** always required the addition of chloroform to the methanolic reaction mixture, since in pure methanol no reductive amination of **1a**–**1c** was observed at all. Instead, even upon the addition of NaBH_4_, the reaction mixture remained a yellow suspension where starting material **1a**–**1c** was isolated by filtration, without any notable conversion. So far, this unusual resistance of **1a**–**1c** to the presence of NaBH_4_ in methanolic solutions can only be explained by the very low solubility of the compounds in methanol. Upon changing the reaction medium to methanol/chloroform (1/1, v/v) the reductive amination of **1a**–**1c** occurred rapidly, as the yellow reaction mixture quickly turned colourless. Contrastingly, the reductive amination of the ethoxy-bearing compounds **1d**–**1f** with NaBH_4_ occurred readily in pure methanol, not requiring the addition of chloroform.

The synthesis trials using aldehydes **g** and **h** were undertaken to reveal whether the presence or lack of a hydroxy group (which is lacking entirely in **g** and is attached in *para* instead of *ortho* position in **h**) on the aromatic moieties had any influence regarding the preferred product ratios of **2**, **3** and **4**. It is noteworthy that after a reductive amination of **1g**, employing the ‘single addition’ procedure, i.e. solely NaBH_4_ addition, the monoalkylated product **4g** could be isolated in notable quantities, whereas after the reduction of **1a**–**1f** the monoalkylated products were commonly detected in low percentages. This observation may indicate that the lack of an aromatic hydroxy group may tend to promote complete hydrolysis of the aminal bridge under reductive amination conditions, thereby favouring **4g** formation and decreasing **3g** and **2g** formation. Nevertheless, both **3g** and **2g** were detectable in the reaction mixture via LC-MS, and the percentage of **2g** could be increased by using the multi-addition procedure (electronic supplementary material), just as was the case for the other substitution patterns **a**–**f** and **h**. This implies that a hydroxy group is not a necessary prerequisite for the successful formation of trialkylated DAZA compounds **2**. Also, the isolation of substantial amounts of **4g** as described herein employed acidification during the work-up process. It has been previously observed, that trialkylated DAZA compounds are prone to *N*-dealkylation under acidic conditions [13]. Thus, **4g** may have formed in excess during the work-up owing to dealkylation of **2g** and **3g**.

Furthermore, the formation of trialkylated DAZA compounds also occurs when using 4-hydroxy-carrying compounds instead of 2-hydroxy-carrying compounds, as was shown for the reductive amination of vanillin-based variant **1h**, which resulted in a mixture of **2h**, **3h** and **4h**, whereby **2h** again could be isolated using the multi-addition procedure. In summary, unambiguous differences in the reductive amination reaction behaviour between the compounds of substitution patterns **a**–**h** were not observed decisively.

### 3.4. Quantum chemical calculations: direct reductive amination of trialkylated compounds, using 3e as representative

Using DFT calculations, the reaction mechanism of the formation of trialkylated compound **2** was investigated. The most obvious and likely reaction pathway is that of a direct reductive amination of the dialkylated compound **3** (figure 4). Herein, a secondary endocyclic nitrogen atom attacks a free carbonyl via a nucleophilic route, leading to the reductive amination, which may occur via either hemiaminal or iminium formation (figure 4) [17].

The barrier height for this reaction pathway was calculated representatively for the **3e** → **2e** turnover and was determined at 138 kJ mol^−1^. Therefore, it notably exceeds the maximum barrier height of 100 kJ mol^−1^ for reactions that still proceed at RT with an appreciable reaction rate [31]. Contrastingly to the QCC thus indicating the reaction pathway to be unlikely, the results from the synthesis trials using the described ‘multi-addition’ procedure verify that the turnover of **3** into **2** indeed occurs, when sufficient carbonyl component is added to the reaction.

### 3.5. Quantum chemical calculations: carbonyl insertion into the aminal bridge?

Notwithstanding the definite occurrence of the direct reductive amination reaction turning compounds **3** into **2** through carbonyl addition to a secondary endocyclic nitrogen atom (figure 4), we asked whether it might be possible that trialkylated compounds **2** are formed from **1**, directly. In this scenario, it would not be necessary for **1** to be reduced to **3** in the first step, but instead, the carbonyl attack may happen at the aminal bridge of **1**. A mechanism for such a straightforward reaction **1** → **2** was previously proposed, featuring a carbonyl insertion into the bisaminal bridge, comparable to observations previously made by Greiser *et al*. [1] and Denat *et al.* [32].

**Figure 5.**
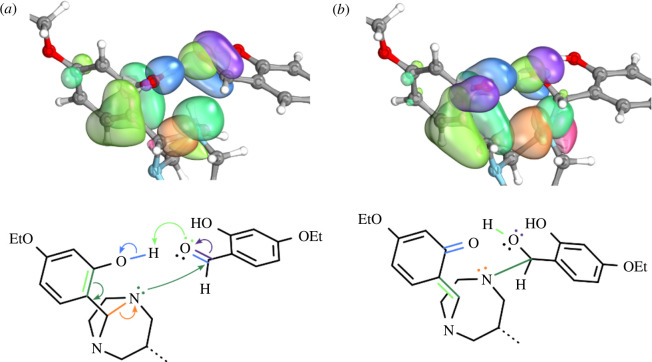
Visualization of the electron flow during the initial attack of a carbonyl unit at **1e** (*a*), towards the formation of transition state **TS 2.1**, in terms of IBOs and curly arrow notation. (*b*) The increasing electrophilicity of the carbonyl carbon owing to the declining carbonyl CO double bond character, making it prone to nucleophilic attack from the aminal nitrogen.

**Figure 6.**
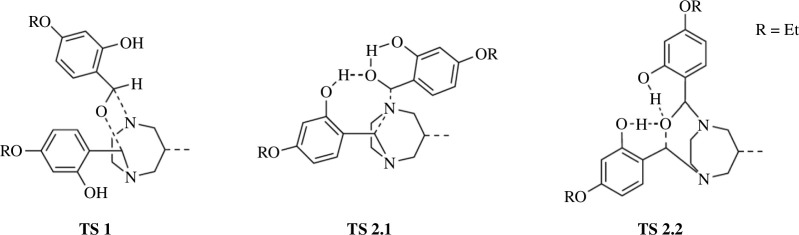
Structures of transition state **TS 1**, resulting from direct carbonyl insertion, but with the hydroxy groups facing away from the reaction centre, and structures of transition states **TS 2.1** and **TS 2.2**, which instead feature hydroxy group interaction via hydrogen bond formation and subsequent hydrogen transfer onto the attacking aldehyde.

Insertion of an alkoxy-salicylaldehyde into the aminal bridge of **1e** was investigated for two conformers of **1e**, differing in the orientation of the 2-hydroxy group towards the attacking aldehyde. In one case, the 2-hydroxy group directly faces the attacking aldehyde and the carbonyl insertion proceeds via **TS 2.1** (figures 5 and 6). In the other case, the 2-hydroxy group faces in the opposite direction, diametrically away from the attacking aldehyde and the reaction proceeds via **TS 1** (figure 6).

Regardless of the orientation of the 2-hydroxy group, the insertion reaction would be initiated by an electrophilic attack of an aminal nitrogen at the carbonyl carbon of the attacking alkoxy-salicylaldehyde. The electron flow during the first part of the reaction via **TS 2.1** is visualized in figure 5 in terms of IBOs and curly arrow notation. When the 2-hydroxy group faces the carbonyl oxygen the hydroxy hydrogen can initially interact with the carbonyl oxygen by means of hydrogen bonding, leading to an increased bond order between both atoms. Simultaneously, the double bond between the carbonyl oxygen and carbonyl carbon is weakened, leading to an increased electrophilicity of the carbonyl carbon. The declining carbonyl C=O double bond character is evident from figure 5: as the reaction progresses towards **TS 2.1**, the π_CO_-IBO becomes a lone-pair p-IBO at the former carbonyl oxygen. The increased electrophilicity at the carbonyl carbon facilitates the nucleophilic attack of the aminal nitrogen. A simplified structure of the resulting transition state **TS 2.1** is shown in figure 6. At **TS 2.1**, the distance between the (hydroxy) hydrogen towards the hydroxy oxygen (124 pm) is actually longer than the distance towards the carbonyl oxygen (115 pm), blurring the distinction between hydroxy and carbonyl oxygens. The partial double bond character between the former hydroxy oxygen and the phenyl ring it is bonded to is also displayed in figure 5. **TS 2.1** is further stabilized by an intramolecular hydrogen bonding interaction between the former carbonyl oxygen and the hydroxy group on the attacking aldehyde (figure 6).

The barrier height between the educts and **TS 2.1** is 117 kJ mol^−1^. Reaction towards **2e** via **TS 2.1** would then continue via a second transition state **TS 2.2** (figure 6). In this second step, the former carbonyl oxygen attacks the aminal carbon, resulting in CO insertion into the aminal bridge. The hydrogen that was transferred from the hydroxy oxygen onto the carbonyl oxygen is transferred back, restoring the hydroxy group. The barrier height (41 kJ mol^−1^) of this second step is much lower compared with the barrier height of the first step, making this second step highly likely at RT.

In contrast to this two-step carbonyl insertion, a one-step insertion reaction path is obtained via **TS 1**, when the hydroxy group faces away from the attacking aldehyde (figure 6). The associated barrier height of the one-step reaction is prohibitively high (245 kJ mol^−1^), preventing this reaction from proceeding at RT. The difference in barrier heights of **TS 2.1**/**TS 2.2** versus **TS 1** (117 and 41 kJ mol^−1^ versus 245 kJ mol^−1^) highlights the importance of the 2-hydroxy group orientation for this hypothetical reaction pathway. The hydrogen bonding interaction and subsequent hydrogen transfer between the 2-hydroxy and carbonyl group greatly stabilize the resulting transition state **TS 2.1**. When the hydroxy group faces away, these interactions are impossible, resulting in the high-energy transition state **TS 1**.

## 4. Discussion

The reductive amination of 1,5-diazabicyclo[3.2.1]octanes **1a**–**1h** with NaBH_4_ leads to the formation of three different products in varying percentages. Among these, the formation of the trialkylated DAZA compounds **2a**–**2f/2h** is probably the most unexpected outcome, because it requires a reductive amination involving the simultaneous addition of a third alkoxy-2-hydroxybenzyl moiety. This reaction occurs even when only NaBH_4_, and no additional alkylating agent or carbonyl component, is added to **1** (single addition). In contrast, the formation of the dialkylated compounds **3a**–**3h** is the far more obvious result, as it likely occurs via reductive cleavage of the aminal bridge (as described with NaBH_4_ for example in previous work [33–35]) and simultaneous reduction of the exocyclic imine bond. Furthermore, the formation of monoalkylated compounds **4**, bearing the remaining hydroxybenzyl moiety at the exocyclic nitrogen, logically proceeds via complete hydrolysis of the aminal bridge, leaving only the exocyclic imine group for reduction. However, in the case of **4a**–**4f**, HPLC analysis often exhibited two close peaks assigned to the monoalkylated substrate (figure 2). This indicates that monoalkylated isomers bearing the remaining hydroxybenzyl moiety at the endocyclic nitrogen may be formed as well, which in turn indicates hydrolysis of either the aminal bridge or the imine bond as the source of a carbonyl compound released *in situ*. The third carbonyl compound, which is required for the formation of trialkylated **2**, may thus stem from both hydrolysis sites. The formation of aldehydes via *in situ* hydrolysis has previously been verified by us [1]. We showed that combining **1b** (4-methoxy) and **1e** (4-ethoxy) in methanol results in a crossover of the carbonyl moieties, giving compounds **1** of mixed substitution pattern (4-methoxy/4-ethoxy), which can only be explained by *in situ* aldehyde release and re-condensation [1].

In the absence of additional eq. of aldehyde, at least one molecule **1** needs to be partially or completely hydrolyzed to produce the *in situ* carbonyl moiety attacking a second molecule **1**, implying a theoretical maximum yield of **2** of 66%. That means hydrolyzed, i.e. monoalkylated or even non-alkylated DAZA, compounds are necessary by-products of the reaction procuring compounds **2**.

We investigated the reductive amination reaction for 2-hydroxy-bearing species (**a**–**f**), non-hydroxy-bearing species **g** and 4-hydroxy-bearing species **h**, to identify possible dependencies of the reaction outcome (i.e. percentages of **2**, **3** and **4**) on the aromatic substitution pattern. So far, no clear differences were observed, indicating that the presence and position of a phenolic hydroxy group are no necessary prerequisite for the formation of trialkylated compounds **2**. Neis [19] has previously reported the synthesis of dialkylated as well as trialkylated DAZA compounds comparable to **2** and **3**, including DAZA compounds bearing pyridine units instead of hydroxybenzyl moieties.

The most obvious reaction pathway resulting in the formation of **2** is a direct reductive amination at the endocyclic secondary nitrogen atom of the dialkylated compounds **3** (figure 4) [16,17]. This was verified by synthesis trials employing the multi-addition procedure described herein, which repeatedly adds a carbonyl component and NaBH_4_ to a dialkylated DAZA species **3**, allowing monitoring of the continuous turnover into trialkylated **2**. This procedure logically provided the trialkylated DAZA compounds in yields higher than the theoretical 66% and allowed for selective isolation of compounds **2**. It is noteworthy that this method reproducibly gave compounds **2a**–**2h**, meaning the additional reductive *N*-alkylation only took place at the endocyclic nitrogen atom. In theory, the exocyclic nitrogen atom also could undergo reductive *N*-alkylation, which means that theoretically, even tetra-alkylated DAZA compounds might be possible. So far, we have not observed such components in any of our synthesis trials, but the possibility of producing such compounds merits further investigation.

Contrastingly to the synthesis trials outcome, QCC of the associated barrier height for direct reductive amination of **3** revealed an activation energy of 138 kJ mol^−1^ (figure 4), meaning it is too high to make its occurrence likely at RT. Nonetheless, small energy errors in QCC can lead to big differences in reaction rates. It must also be noted that solvent influences may not have been sufficiently taken into account in those calculations. The calculations presented herein were performed under the assumption of a methanolic solution. However, the reductive amination of the methoxy-bearing compounds **1a**–**1c** and **1h** was performed using a mixture of methanol and chloroform (1/1, v/v), since there was no distinct turnover observed when pure methanol was used. The choice of solvent could influence the specificity of the reaction regarding the formation of competing products **2** and **3**, since particularly methanol likely partakes in activation or intermediate stabilization via proton donation or hydrogen bridge bonding. Furthermore, the reaction might be susceptible to other parameters, like content of water or catalytic influences of trace metals. These parameters are not yet reflected in the QCC, which may explain the gap between low reaction occurrence in theory and the obvious reaction occurrence as verified by the synthesis trials (multi-addition).

An alternative mechanism explaining the formation of trialkylated **2** directly from **1** was explored via QCC, but so far remains entirely hypothetical as no corresponding intermediate has so far been observed and isolated experimentally (figures 5 and 6). During this hypothetical reaction pathway, the involvement of the 2-hydroxy group lowers the activation energy barrier of a carbonyl insertion into the N–C–N aminal bridge from 245 (**TS 1**) to 117 kJ mol^−1^ (**TS 2.1**) by means of hydrogen bond formation and stabilization of the transition state (figures 5 and 6). Such a carbonyl insertion into the N–C–N aminal bridge could follow a two-step mechanism via the transition states **TS 2.1** and **TS 2.2**. According to the Eyring equation [36], appreciable reaction rates at RT are observed for barriers up to 100 kJ mol^−1^ [31]. The calculated barrier height of 117 kJ mol^−1^ for the formation of **TS 2.1** is slightly higher, corresponding to a small reaction rate. The results from this calculation would indicate that compounds lacking a 2-hydroxy group cannot undergo the carbonyl-amine insertion reaction and thus not form trialkylated DAZA species. Since our synthesis trials have proven otherwise, all in all this alternative insertion mechanism seems to be of little importance for the actual reaction outcome, and must be considered purely hypothetical.

## 5. Conclusions

The formation of trialkylated DAZA compounds **2** is accompanied by the formation of two major byproducts, namely the dialkylated compounds **3** and the monoalkylated compounds **4**. A method employing multiple addition of aldehyde component and NaBH_4_ allows one to shift the reaction towards the preferential formation of **2**, thus enabling the isolation of new potential radiometal chelators based on the *N*,1,4-tri(alkoxy-2-hydroxybenzyl)-DAZA scaffold, serving as precursor for promising radiotracers in nuclear medicine.

## Data Availability

All data (i.e. the chemical characterization of the described compounds) are provided in electronic supplementary material [37].
